# DNA Shuffling of *aprE* Genes to Increase Fibrinolytic Activity and Thermostability

**DOI:** 10.4014/jmb.2202.02017

**Published:** 2022-04-25

**Authors:** Zhuang Yao, Hye Sung Jeon, Ji Yeon Yoo, Yun Ji Kang, Min Jae Kim, Tae Jin Kim, Jeong Hwan Kim

**Affiliations:** 1 Division of Applied Life Science (BK21 4), Graduate School, Gyeongsang National University, Jinju 52828, Republic of Korea; 2 Institute of Agriculture and Life Science, Gyeongsang National University, Jinju 52828, Republic of Korea

**Keywords:** DNA shuffling, *aprE*, *Bacillus subtilis*, fibrinolytic enzymes

## Abstract

Four *aprE* genes encoding alkaline serine proteases from *B. subtilis* strains were used as template genes for family gene shuffling. Shuffled genes obtained by DNase I digestion followed by consecutive primerless and regular PCR reactions were ligated with pHY300PLK, an *E. coli*-*Bacillus* shuttle vector. The ligation mixture was introduced into *B. subtilis* WB600 and one transformant (FSM4) showed higher fibrinolytic activity. DNA sequencing confirmed that the shuffled gene (*aprEFSM4*) consisted of DNA mostly originated from either *aprEJS2* or *aprE176* in addition to some DNA from either *aprE3-5* or *aprESJ4*. Mature AprEFSM4 (275 amino acids) was different from mature AprEJS2 in 4 amino acids and mature AprE176 in 2 amino acids. *aprEFSM4* was overexpressed in *E. coli* BL21 (DE3) by using pET26b(+) and recombinant AprEFSM4 was purified. The optimal temperature and pH of AprEFSM4 were similar to those of parental enzymes. However, AprEFM4 showed better thermostability and fibrinogen hydrolytic activity than the parental enzymes. The results indicated that DNA shuffling could be used to improve fibrinolytic enzymes from *Bacillus* sp. for industrial applications.

## Introduction

*Bacillus* species such as *B. subtilis*, *B. amyloliquefaciens* and *B. licheniformis* secrete several proteases, and AprE, an alkaline protease, is the most important of these enzymes [1]. Some AprEs possess strong fibrinolytic activities, and nattokinase, secreted by some *B. subtilis* strains, is the most well-known example. Nattokinase is available on the market as a nutraceutical supplement [2]. AprEs with strong fibrinolytic activities are considered a promising alternative for medically important thrombolytic agents such as tPA (tissue plasminogen activator), urokinase, and streptokinase [3]. Compared with these thrombolytic agents, *Bacillus* enzymes have several advantages including convenient intake via the oral route, longer half-lives inside the body, and fewer serious side effects such as bleeding [2]. In addition, *Bacillus* enzymes are GRAS (generally recognized as safe) compounds and can be easily produced on a large scale economically [4]. *Bacillus* species secreting fibrinolytic enzymes similar to nattokinase have been isolated from traditional Korean fermented foods including doenjang (soy paste), ganjang (soy sauce), cheonggukjang (fermented soybean), and jeotgal (fermented seafood) [5-8]. These enzymes not only show high homologies with nattokinase in their amino acid sequence but also possess high fibrinolytic activities [7]. Moreover, *Bacillus* fibrinolytic enzymes from traditional Korean fermented foods have great potential as an alternative to the currently used thrombolytic agents. If the fibrinolytic activities and/or stabilities of these enzymes are improved further, their potential for industrial application will likely increase in kind.

In vitro evolution of a gene is the method of choice for improving the properties of its protein product. Meanwhile, family gene (DNA) shuffling is the most effective method for obtaining diverse mutants through extensive recombination between homologous genes [9]. Compared with error-prone PCR (EP-PCR), DNA shuffling is a more powerful technique, generating various chimeras by random recombination between homologous sequences [10]. The advantages of DNA shuffling stem from its ability to generate diverse mutants because recombination can occur between many regions of homologous genes, which is not possible by EP-PCR. DNA shuffling has been successfully used to improve the activity of a urate oxidase [11], the thermostability of an endo-β-1,4-glucanase [12] and a xylanase [13].

In this study, DNA shuffling was used on 4 *aprE* genes from *B. subtilis* strains isolated from traditional Korean fermented foods to obtain mutants capable of producing more active or stable fibrinolytic enzymes, which will benefit the industrial application of the *aprE* genes.

## Materials and Methods

### Bacterial Strains and Plasmids

Bacterial strains and plasmids used in this study are shown in Table 1. *Bacillus* and *E. coli* were grown in lysogeny broth (LB, 10 g peptone, 5 g yeast extract, 5 g NaCl per liter, pH 7.0) at 37°C with aeration. *Bacillus subtilis* harboring pHY300PLK (4.87 kb, Ap^r^, Tc^r^) or pHYFSM4 (pHY300PLK with *aprEFSM4*) was cultivated in LB with tetracycline (Tc, 10 μg/ml). *E. coli* containing pET-26b(+) (Merck Millipore, Germany, 5.4 kb, Km^r^) or pETFSM4 (pET-26b(+) with *aprEFSM4*) was cultivated in LB with kanamycin (Km, 30 μg/ml).

### Template Genes and Family Gene Shuffling

Four *aprE* genes were used as templates for family gene shuffling (Table 1), namely, *aprE3-5* (same as aprE2, DQ997812) from *B. subtilis* CH3-5 [7, 15], *aprEJS2* (MF677779) from *B. subtilis* JS2 [16], *aprESJ4* (MK796246) from *B. subtilis* SJ4 [17], and *aprE176* (KJ572414) from *B. subtilis* HK176 [18]. These genes were previously cloned into pHY300PLK, a *Bacillus*–*E. coli* shuttle vector, and the plasmids were used as the templates for PCR amplification of the *aprE* genes. For amplifying *aprE3-5*, aprE-F and aprE-R were used: aprE-F (5'-GCGAATTCGCCGCATCTGTGTCTTTG-3') and aprE-R (5'-GCGAATTCGAGAACAGA GAAGCCGCT-3')(EcoRI site underlined). For amplifying other *aprE* genes, CH51-F and CH51-R were used: CH51-F (5’-AGGATCCCAAGAGAGCGATTGCGGCTGTGTAC-3’, BamHI site underlined) and CH51-R (5’-AGAATTCTTCAGAGGGAGCCACCCGTCGATCA-3’, EcoRI site underlined) [16]. PCR was done using the MJ Mini Personal Thermal Cycler (BioRad, USA). The reaction mixture (50 μl) contained 1 μl template DNA, 2 μl each primer (10 μM), 1 μl dNTP (0.25 mM), and 0.5 μl Ex*Taq* DNA polymerase (Takara, Japan). Amplified *aprE* genes were purified by using a PCR clean-up kit (Favorgen, Taiwan).

One microgram from each *aprE* gene was mixed together and digested by DNase I (0.3 U, Sigma-Aldrich, USA) in 50 mM Tris–HCl (pH 7.4) and 10 mM MgSO_4_ [10]. During 30 min digestion at 15°C, aliquots were taken every 5 min and the degree of digestion was examined by agarose gel (2%, w/v) electrophoresis. The 1^st^ PCR (primerless PCR) was carried out with the digested fragments as templates. The reaction mixture (50 μl) consisted of 1 μg DNA, 0.25 mM each dNTP, and 2.5 U Ex*Taq* polymerase (Takara). The 1^st^ PCR was done under the following conditions: initial denaturation at 95°C for 4 min followed by 40 cycles of 95°C for 30 s, 60–42°C (3°C interval) for 30 s, and 72°C for 2 min, and the final extension at 72°C for 7 min. The 2^nd^ PCR was done with 5 μl of the 1^st^ PCR product as the template, 2 μl each primer (10 pmol), 5 μl dNTPs (0.25 mM), and 0.5 μl Ex*Taq* DNA polymerase (Takara). CH51-F and CH51-R were used as the primers.

### Screening of Mutants

The 2^nd^ PCR products were purified from an agarose gel, digested with BamHI and EcoRI, and ligated with pHY300PLK. The ligation mixture was used to transform *B. subtilis* WB600 competent cells by electroporation. Transformants (TFs) were obtained on LB agar plates (Tc, 10 μg/ml) incubated for 24 h at 37°C. The TFs were spotted on LB skim milk (1%, w/v) plates which were then incubated at 37°C for 48 h. Colonies showing higher proteolytic activities than control were selected, and their fibrinolytic activities were measured by the fibrin plate method. *B. subtilis* WB600 carrying pHY3-5 (pHY300PLK with *aprE3-5*) was used as the control.

Preparation of *B. subtilis* WB600 competent cells and electroporation (200 Ω, 18 kV/cm) were conducted according to the previous report [16]. Plasmid DNA preparation, restriction enzyme digestion, and agarose gel electrophoresis were performed according to published methods [19].

### Overexpression of *aprEFSM4* in *E. coli* BL21 (DE3) and Purification of AprEFSM4

*aprEFSM4* without its signal sequence was amplified by using a primer pair: pET-F (5’-AGAGGATCCGAT GGCAGGGAAATCA-3’, BamHI site underlined) and pET-R (5’-AGACTCGAGCTGAGCTGCCGCCTG-3’, XhoI site underlined). PCR conditions were as follows: 94°C for 5 min followed by 30 cycles consisting of 94°C for 30 s, 60°C for 30 s, and 72°C for 1 min. The amplified fragment was inserted into pET26b(+) (Merck Millipore, Germany) after being digested with BamHI and XhoI, resulting in pETFSM4. *E. coli* BL21 (DE3) competent cells were transformed with pETFSM4 by electroporation. An *E. coli* BL21 (DE3) TF harboring pETFSM4 was grown in LB (250 ml) containing Km (30 μg/ml) until the OD_600_ reached 0.8. Then, IPTG (isopropyl β-D-1-thiogalactopyranoside) was added (1 mM), and the culture was incubated for 20 h at 30°C. The cell pellet was obtained by centrifugation and resuspended in 5 ml phosphate-buffered saline (PBS). Cells were disrupted by sonication (5 cycles of 1 min sonication with 2 min cooling) using a sonicator (UW 2070, Bandelin, Germany), and the cell extract was centrifuged at 12,000 ×*g* for 15 min at 4°C. The resulting supernatant (soluble fraction) and pellet (insoluble fraction) were examined by SDS-PAGE after the insoluble fraction was resuspended with binding buffer (20 mM sodium phosphate, 0.5 M NaCl, 10 mM imidazole, pH 7.4). AprEFSM4 was purified from the soluble fraction by using a HiTrap IMAC FF column (GE Healthcare, Sweden). The imidazole concentration of elution buffer (20 mM sodium phosphate, pH 7.4, imidazole, 0.5 M NaCl) was increased from 100 mM to 500 mM at 100 mM increments to elute bound AprEFSM4 from the column. One microliter from each fraction (1 ml) was spotted onto a fibrin plate and the plate was incubated at 37°C. Active fractions were pooled, dialyzed against 20 mM sodium phosphate buffer (pH 7.4) for 24 h, and concentrated by using an Amicon filter (MWCO 12,000; Merck Millipore).

### Properties of Recombinant AprEFSM4

The effect of pH on the AprEFSM4 was examined at pH 3–11 by using citrate-NaOH (pH 3–5), sodium phosphate (pH 6–8), and Tris-HCl (pH 9–11) buffers (all 50 mM). AprEFSM4 (1 μg) in each buffer was incubated for 1 h at 40°C and then the fibrinolytic activity was measured by the fibrin plate method. AprEFSM4 (1 μg) in each buffer was incubated for 6 h at 40°C and the activity was measured at 1, 3, and 6 h. AprEFSM4 (1 μg) in sodium phosphate buffer (pH 8) was incubated for 30 min at 37-60°C and the activities were measured. AprEFSM4 (1 μg) in sodium phosphate buffer (pH 8) was incubated for 3 h at 37-60°C and the activity was measured at 0.5, 1, 2, and 3 h. AprEFSM4 was exposed to 5 mM metal ions or 1 mM inhibitors for 30 min at 40°C (pH 8), and then the remaining activities were measured.

### Amidolytic Activity

The amidolytic activity of AprEFSM4 was measured by using N-succinyl-ala-ala-pro-phe-p-nitroanilide (S7388, Sigma-Aldrich, USA) as the substrate [16]. Vmax and Km values were determined by measuring the hydrolysis rates at different substrate concentrations ranging from 0.03 to 0.9 mM. *K*cat was determined from the relationship, *K*cat = *V*max/[enzyme].

### Hydrolysis of Fibrinogen

Fibrinogen (1 mg, bovine) (MP Biochemicals, IIIkirch-Graffenstaden, France) was mixed with AprEFSM4 (50 ng) and the mixture in 1 ml of 20 mM Tris-HCl (pH 8.0) was incubated at 37°C up to 12 h. Aliquots were taken at intervals and mixed with a 5 × SDS sample buffer. After boiling for 5 min, samples were analyzed by SDS-PAGE [18].

## Results and Discussion

### DNA Shuffling of DNase I-Digested *aprE* Genes

Four *aprE* genes from *B. subtilis* strains were selected as templates for family gene shuffling because they all confer high fibrinolytic activity to the host cells. AprE176 and AprEJS2 show 98.4% amino acid sequence homology with each other, but both enzymes show 86% homologies with AprE3-5 and AprESJ4. It was expected that the significant amino acid sequence differences between AprE176 group (AprE176 and AprEJS2) and AprE3-5 group (AprE3-5 and AprESJ4) might generate diverse recombinants via family gene shuffling.

**Fig. 1:**
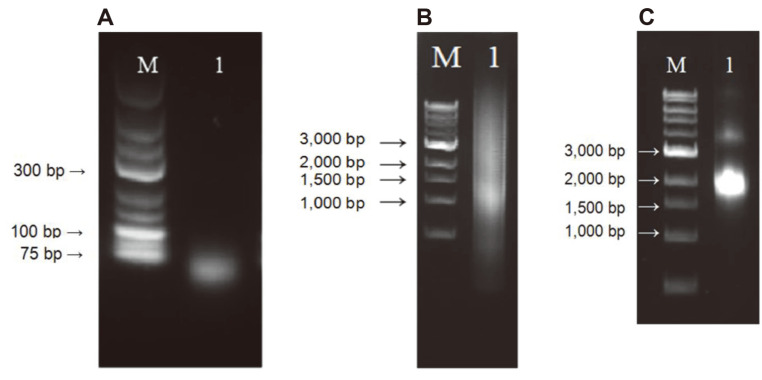
DNase I digestion of 4 *aprE* templates and reassembly from digested DNA by PCR. (**A**) M, GeneRuler low range DNA ladder (SM1193, Thermoscientific); 1, *aprE* genes after DNase I (0.3 unit) digestion for 25 min at 15°C. Agarose gel (2%, w/v) was used. (**B**) M, 1 kb DNA ladder (N3223, New England Biolabs, USA); 1, 1^st^ PCR product. (**C**) M, 1kb DNA ladder (N3223); 1, 2^nd^ PCR product. PCR products were analyzed by agarose gel (1%) electrophoresis.

As DNase I digestion time increased, the portion of small-sized DNA fragments increased. Distinct *aprE* bands disappeared after 10 min, and most DNAs were 100–150 bp in size after 20 min (results not shown). All DNAs were less than 100 bp after 30 min. Based on these observations, 4 *aprE* genes were digested for 25 min at 15°C and most DNAs were 75–100 bp in size under that condition (Fig. 1A). An agarose gel slice containing 75–100 bp DNA was excised and the DNA was purified by using a gel extraction kit (Favorgen, Taiwan) and concentrated by ethanol precipitation. The 1^st^ PCR was done without primers, and products around 1.5 kb in size were obtained (Fig. 1B). The 2^nd^ PCR, on the other hand, was carried out with a primer pair and a band around 1.8 kb in size was observed after 15 cycles (Fig. 1C). An agarose gel slice containing the 1.8 kb band was excised and the DNA was purified. The purified DNA was digested with BamHI and EcoRI, and ligated with pHY300PLK. The ligation mixture was used to transform *B. subtilis* WB600 competent cells.

### Screening of Transformants Showing Increased Fibrinolytic Activities

A total of 524 *B. subtilis* WB600 TFs were examined for the proteolytic activities on LB skim milk plates. One TF, FSM4, showed a larger hydrolytic zone than control, and the TF showed 12.76% higher fibrinolytic activity than control, *B. subtilis* WB600 carrying pHY3-5 (pHY300PLK with *aprE3-5*), when the fibrinolytic activities of both strains were compared by the fibrin plate method. The nucleotide sequence of *aprEFSM4* was determined including its promoter sequences (1,248 nt) and compared with template genes (Table 2, Fig. S1). Sequence analyses showed that most parts of *aprEFSM4* originated from either *aprEJS2* or *aprE176* with a few fragments originated from either *aprE3-5* or *aprESJ4*. The -35 (TCTACT, 8–13 nt in Fig. S1) and -10 (TACAAT, 33-38 nt) promoter sequences were conserved among all 4 templates and *aprEFSM4*. However, CTAT (29–32 nt) and G (53 nt) in the regulatory region of *aprEFSM4* were derived from either *aprE3-5* or *aprESJ4*. *aprEFSM4* showed the highest nucleotide sequence homology with *aprEJS2* (99.12%, 1,138/1,148 bp) followed by *aprE176* (98.61%, 1,132/1,148 bp), but showed lower values with *aprE3-5* (80.05%, 919/1,148 bp) and *aprESJ4* (79.53%, 913/1,148 bp). The results confirmed that most regions of *aprEFSM4* originated from either *aprEJS2* or *aprE176*. Differences in the nucleotide and amino acid sequences are summarized in Table 2. Although most parts of *aprEFSM4* were originated from either *aprEJS2* or *aprE176*, some regions originated from either *aprE3-5* or *aprESJ4* (marked with a box in the Fig. S1). For example, regions around 433, 902, and 977 nt of *aprEFSM4* originated from either *aprE3-5* or *aprESJ4*. The results confirmed that DNA shuffling occurred between all 4 template genes although extensive sequence changes did not occur as expected. This was most likely caused by the high nucleotide sequence homologies among templates, especially between *aprE176* and *aprEJS2* (98% nucleotide sequence identity). Many recombinations probably occurred between fragments derived from *aprE176* and *aprEJS2*, but these recombinations were not noticed due to shared sequences. More diverse mutants could be obtained if *aprE* genes with fewer homologies with each other are used for family gene shuffling. As expected from the nucleotide sequence homologies, AprEFSM4 showed the highest amino acid sequence homology with AprEJS2 and AprE176 (Fig. S2). AprEFSM4 was different from AprEJS2 in 4 amino acids (98.95%, V180A, V254I, G268S, and R363K), and different from AprE176 in 4 amino acids (T26S, S27P, S251A, and G268S). Amino acids of AprEJS2 and AprE176 are shown first. The 268^th^ amino acid of AprEFSM4 was serine, which differed from glycine of AprEJS2 and AprE176, indicating that the corresponding fragment was derived from either *aprE3-5* or *aprESJ4*. AprEFSM4 showed 86.13% (329/382 aa) homology with AprE3-5 and 85.34% (326/382 aa) homology with AprESJ4.

### Overexpression of *aprEFSM4* in *E. coli* and Purification of Recombinant AprEFSM4

**Fig. 2:**
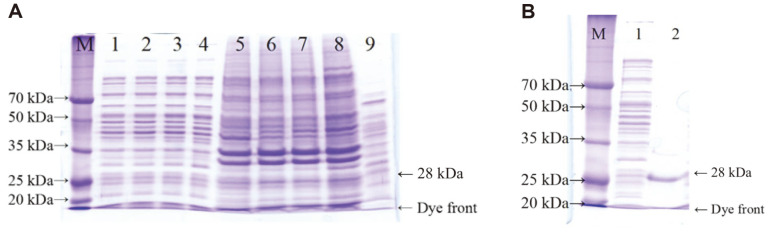
Overproduction (**A**) and purification (**B**) of recombinant AprEFSM4. M, Dokdo-marker broad-range (EBM1034, Elpis Biotech. Korea); 1–4 soluble fraction from *E. coli* cells grown for 2 h (1), 4 h (2), 10 h (3), and 20 h (4) after IPTG induction; 5–8, insoluble fraction from *E. coli* cells grown for 2 h (5), 4 h (6), 10 h (7), and 20 h (8) after IPTG induction; 9, a negative control, soluble fraction from *E. coli* BL21 [pET26b(+)] grown for 20 h without induction. (B). M, Dokdo-marker broad-range; 1, soluble fraction from *E. coli* cells; 2, purified AprEFSM4 from soluble fraction by affinity column chromatography.

Soluble and insoluble fractions were obtained from IPTG-induced *E. coli* BL21 (DE3) [pETFSM4] culture and analyzed by SDS-PAGE. Two thick bands were observed from the insoluble fractions and the apparent size of each band was 35 and 31 kDa, respectively (Fig. 2A). The molecular weight of proAprEFSM4 with 8 extra amino acids at the C-terminus was calculated to be 36,965.36 Da and that of a mature enzyme with 8 extra amino acids was 28,555.75 Da. Considering these, the 2 bands were likely to be pro- and mature form of AprEFSM4. The same bands were also observed from soluble fractions although the band intensities were lower. AprEFSM4 was purified from the soluble fraction since active AprEFSM4 was likely present in the soluble fraction. AprEFSM4 was eluted from the affinity column at 200 mM imidazole concentration and showed fibrinolytic activity on a fibrin plate. The size was 28 kDa, which matched the calculated size of the mature enzyme (Fig. 2B). The results indicated that mature and active AprEFSM4 was produced in *E. coli* although more AprEFSM4 was present in inclusion bodies.

### Properties of Recombinant AprEFSM4

**Fig. 3:**
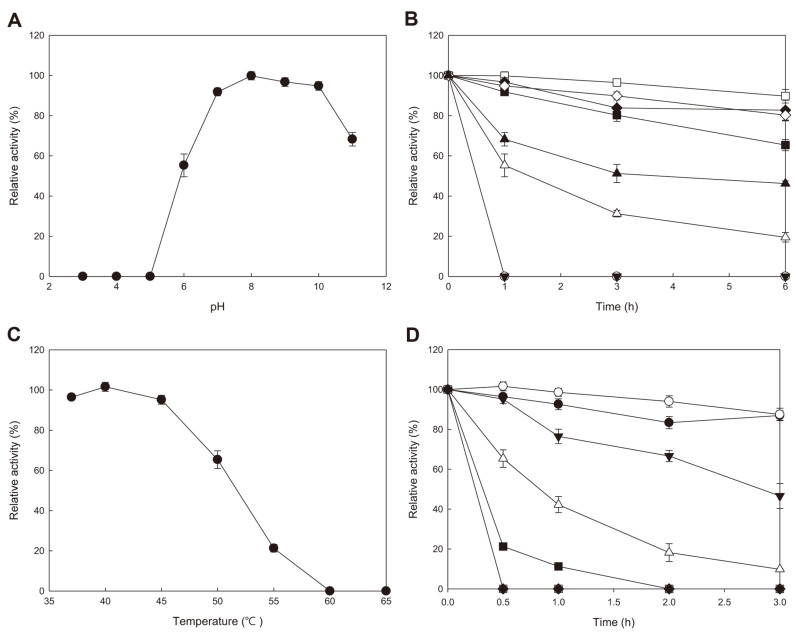
The effect of pH and temperature on the fibrinolytic activity of recombinant AprEFSM4. (**A**) Optimum pH, (**B**) pH stability. -●-, pH 3; -○-, pH 4; -▼-, pH 5; -△-, pH 6; -■-, pH 7; -□-, pH 8; -◆-, pH 9; -◇-, pH 10; -▲-, pH 11. (**C**) Optimum temperature, (**D**) Thermostability. -●-, 37°C; -○-, 40°C; -▼-, 45°C; -△-, 50°C; -■-, 55°C; -□-, 60°C.

AprEFSM4 maintained activity at pH 7–10 and the optimum pH was 8 (Fig. 3A). The relative activity was 89.35, 100, 96.67, and 94.80% at pH 7, 8, 9, and 10, respectively. The activity decreased rapidly at pH 6 (55.28%) and 11 (68.26%), and no activity was detected at pH 5 and below. During 6 h exposure at different pH, the activity was stable for the first hour and then decreased gradually at pH 8 and 9 (Fig. 3B). At pH 8-10, more than 80% of the activity remained after 6 h. At pH 6, 7, and 11, the activity decreased rapidly within the first 3 h and then decreased slowly. At pH 5 and below, the activity decreased rapidly and no activity was detected after 1 h. The optimum temperature was 40°C at pH 8 (Fig. 3C). No activity was detected after 30 min at 60°C (Fig. 3C). During 3 h exposure at 45°C or 50°C, the activity decreased rapidly within the first hour, then became stable, and decreased again during the last hour (Fig. 3D). During 3 h exposure at 37°C and 40°C, the activity gradually decreased, but more than 85% of the activity remained after 3 h (Fig. 3D). The half-lives of AprEFSM4 were calculated to be 9.76, 11.50, 2.87, 0.83, and 0.33 h at 37, 40, 45, 50, and 55°C, respectively.

These results showed that AprEFSM4 possessed moderate thermostability like the template enzymes. The optimum temperature and pH of AprEFSM4 were the same as those of AprEJS2 (40°C, pH 8) [16] and AprE176 (40°C, pH 8) [18], and similar to that of AprE3-5 (40°C, pH 7) [15].

Importantly, AprEFSM4 showed better thermostability than the template enzymes. AprEFSM4 retained 21.26% and 11.27% activity after 30 min and 1 h exposure at 55°C, respectively. On the contrary, AprE3-5, AprEJS2, and AprE176 were completely inactivated after 30 min exposure at 55°C [15, 16, 18]. These template enzymes were prepared following the same method used for AprEFSM4, *i.e.*, they were overproduced in *E. coli* BL21(DE3) by pET26b(+) and purified by affinity chromatography using the same column. AprESJ4 was not purified (no data available). After 3 h exposure at 40°C, AprEFSM4 maintained 87.46% activity whereas AprEJS2 maintained 77.98% activity [16]. The increased thermostability of AprEFSM4 seemed to be the result of recombinations although most parts of *aprEFSM4* were derived from either *aprEJS2* or *aprE176* (Table 2). The detailed 3D structure of AprEFSM4 might be different from those of AprEJS2 and AprE176 because of a few changes in amino acids. Amino acid substitutions in proteins are likely to affect interactions between side groups of amino acids such as hydrogen bonds, hydrophobic interactions, and electrostatic interactions, resulting in changes in thermostability [20]. Even a few changes in the amino acid sequence can result in the increased thermostability of an enzyme. Argos *et al*. reported that mutations, either those increasing the internal hydrophobicity of a protein or those increasing the stability of the helix, increased thermostability [21]. For glucose oxidase from *Penicillium notatum* F4, even a single amino substitution (S100A, D408W) caused a significant increase in thermostability [22]. Future studies are necessary to find out the exact cause of the increased thermostability of AprEFSM4.

The effects of metals and inhibitors on AprEFSM4 were examined (Table 3). The activity of AprEFSM4 was increased by Ca^2+^ (117.24%) and Mg^2+^ (107.33%) but reduced by Fe^3+^ (80.89%), Co^2+^ (79.53%) and Mn^2+^ (82.26%). The activity was completely destroyed by PMSF (phenylmethylsulfonyl fluoride). Only 13.75% and 10.37%activity remained after EDTA (ethylenediaminetetraacetic acid) and EGTA (ethylene glycol tetraacetic acid) treatment, respectively. SDS decreased the activity slightly (90.47%). The results were similar to AprE176 but different from AprE3-5 and AprEJS2. The activity of AprE176 was reduced greatly by EDTA (4.5%) and EGTA (6.6%) [18], while the activity of AprEJS2 was reduced by EDTA (77.5%) and EGTA (64.7%) [16]. Finally, the activity of AprE3-5 was affected slightly by EDTA (98.2%) and EGTA (93.3%) treatment [15].

### Amidolytic Activity of AprEFSM4

The *K*m and *V*max of AprEFSM4 were 0.28 mM and 54.05 μM/min, respectively. The *K*cat was 24.76 S^-1^, and the *K*cat/*K*m was 8.84 × 10^4^ S^-1^ M^-1^. The Km of AprEFSM4 was larger than that of AprEJS2 (0.09 mM) [16] but smaller than those of AprE3-5 (0.559 mM) [15] and AprE176 (0.453 mM) [18]. The *K*cat of AprEFSM4 was larger than those of AprEJS2 (7.66 S^-1^) and AprE3-5 (17.344 S^-1^) but smaller than that of AprE176 (122.851 S^-1^). The catalytic parameter (*K*cat/*K*m) of AprEFSM4 was larger than those of AprEJS2 (8.51 × 10^4^ S^-1^ M^-1^) and AprE3-5 (3.1 × 10^4^ S^-1^ M^-1^) but smaller than that of AprE176 (28.2 × 10^4^ S^-1^ M^-1^). No data are available for AprESJ4. The catalytic efficiency of AprEFSM4 was better than that of AprEJS2 but lower than that of AprE176. The results together with the effects of inhibitors suggested that AprEFSM4 showed intermediate catalytic properties between AprE176 and AprEJS2.

### Hydrolysis of Fibrinogen by AprEFSM4

**Fig. 4:**
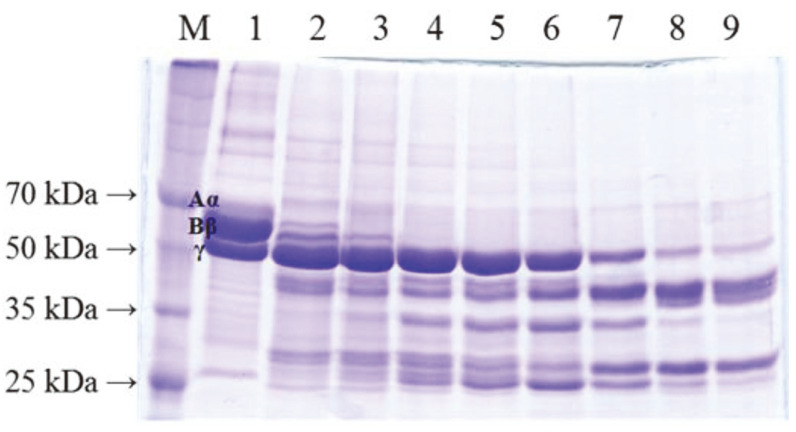
Fibrinogen hydrolysis by recombinant AprEFSM4. M, Dokdo-marker broad-range (EBM^-1^034); 1, control (no enzyme treatment); 2, 5 min; 3, 10 min; 4, 20 min; 5, 30 min; 6, 1 h; 7, 3 h; 8, 6 h; 9, 12 h. A 10% acrylamide gel was used.

Recombinant AprEFSM4 hydrolyzed the Aα and Bβ chains of fibrinogen quickly. The Aα chain was the most sensitive, becoming completely hydrolyzed in 5 min (Fig. 4). The Bβ chain was hydrolyzed within 20 min. However, the γ chain was not completely hydrolyzed even after 12 h. The results indicated that AprEFSM4 had strong α- and β-fibrinogenase activities, and moderate γ-fibrinogenase activity. The pattern was similar to those of the template enzymes. AprE3-5, AprEJS2, and AprE176 showed strong α-fibrinogenase activities together with moderate β-fibrinogenase activities [15, 16, 18]. It took 5 to 10 min for complete hydrolysis of the α-chain, and more than 20 min to 3 h for the β-chain. But γ-chain degradation took longer, exceeding 24 h. AprEFSM4 showed similar α-fibrinogenase activity with its template enzymes but higher β-fibrinogenase activity than AprEJS2 and AprE3-5. The fibrinogenase activities of AprE176 were measured only for the first 20 min. Compared with AprE176, AprEFSM4 has similar α-fibrinogenase activity, and both enzymes completely hydrolyzed the α-chain very quickly. AprEFSM4 showed slightly higher β- fibrinogenase activity than did AprE176 at 20 min of digestion. AprEFSM4 also hydrolyzed the γ-chain more efficiently than either AprEJS2 or AprE3-5 in 12 h. No data on the γ-fibrinogenase activity of AprE176 were available for longer times. These results indicated that AprEFSM4 hydrolyzed fibrinogen more efficiently than did the template enzymes.

In this study, a chimeric gene, aprEFSM4, was obtained from 4 parental *aprE* genes by family gene shuffling. Although most regions of *aprEFSM4* originated from *aprEJS2* and *aprE176*, *aprEFSM4* still contained some regions originated from either *aprESJ4* or *aprE3-5*. AprEFM4 showed better thermostability at 55°C than its parental enzymes. Presumably, some amino acid substitutions might confer better thermostability on AprEFSM4. Higher thermostability for AprEFSM4 is desirable when the enzyme or its producing host cells are included in foods or medicines that might be exposed to the processing at high temperatures.

AprEFSM4 also showed improved fibrinogen hydrolytic activity, also a sought-after improvement for fibrinolytic enzymes and their applications. Moreover, further improvements seem feasible if template genes with fewer homologies with each other are used for family gene shuffling. Family gene shuffling is an efficient method for obtaining diverse mutants, and thus seems an effective alternative for screening samples from various natural environments to isolate microorganisms with strong fibrinolytic activities [23, 24]. To sum up, *aprEFSM4* has good potential as a source for the mass production of fibrinolytic enzymes for the food and pharmaceutical industries.

## Supplemental Materials

Supplementary data for this paper are available on-line only at http://jmb.or.kr.

## Figures and Tables

**Table 1:** Bacterial strains and plasmids used in this study.

Strains, plasmids, or genes	Description	References
*Bacillus subtilis* WB600	Mutant lacking 6 extracellular proteases	[14]
*Escherichia coli* BL21 (DE3)	A host for gene overexpression, carrying T7 RNA polymerase gene	Novagen
pHY300PLK	4.8 kb, *Escherichia coli*–*Bacillus subtilis* shuttle vector. Tc^r^, Am^r^.	Takara
pET26b(+)	5.4 kb, T7 promoter, Km^r^	Novagen
*aprE3-5*	A fibrinolytic gene from *B. subtilis* CH3-5	[7, 15]
*aprEJS2*	A fibrinolytic gene from *B. subtilis* JS2	[16]
*aprESJ4*	A fibrinolytic gene from *B. subtilis* SJ4	[17]
*aprE176*	A fibrinolytic gene from *B. subtilis* HK176	[18]
*aprEFSM4*	A shuffled gene from 4 *aprE* genes	This study

**Table 2:** Nucleotide sequences of *aprEFSM4* and template genes.

Nt	FSM4	JS2	176	3-5	SJ4	Originated from
176	T	T	A (T26S)*	T	T	JS2
179	C	C	T (S27P)	X	X	JS2
190	G	G	T	T	T	JS2
220	C	C	T	C	C	JS2 or 3-5 or SJ4
235	A	A	G	A	A	JS2 or 3-5 or SJ4
262	T	T	C	C	C	JS2
355	C	C	T	T	T	JS2
433	T	G	G	T	T	3-5 or SJ4
562	A	G	A	A	A	176 or 3-5 or SJ4
639	C	T (V180A)	C	C	C	176 or 3-5 or SJ4
851	G	G	T (S251A)	T	T	JS2
860	A	G (V254I)	A	A	A	176 or 3-5 or SJ4
871	A	A	T	T	T	JS2
902	A	G (G268S)	G (G268S)	A	A	3-5 or SJ4
922	C	C	T	C	C	JS2 or 3-5 or SJ4
955	G	G	C	T	T	JS2
977	A	C	C	A	A	3-5 or SJ4
985	T	A	T	A	A	176
1054	A	G	A	A	A	176 or 3-5 or SJ4
1060	A	A	G	T	T	JS2
1117	T	C	T	G	G	176
1188	A	G (R363K)	A	A	A	176 or 3-5 or SJ4

*Amino acid change due to DNA shuffling. Amino acid of AprJS2 or AprE176 was shown first and that of AprEFSM4 later. The number is the location of the amino acid.

**Table 3:** Effects of metal ions and inhibitors on the activity of AprEFSM4.

Metal ion (5 mM)	Relative activity (%)	Inhibitor (1 mM)	Relative activity (%)
None	100	PMSF	0.00 ± 0.00
Mn^2+^	82.26 ± 1.15	EDTA	13.75 ± 1.42
Mg^2+^	107.33 ± 2.67	EGTA	10.37 ± 0.67
Ca^2+^	117.24 ± 1.78	SDS	90.47 ± 1.01
K^+^	99.04 ± 0.13		
Zn^2+^	95.51 ± 2.21		
Fe^3+^	80.89 ± 1.67		
Na^+^	100.51 ± 2.47		
Co^2+^	79.53 ± 2.68		

The counterion for the tested metals was chloride.

All values are the mean ± SD (*n* = 3).
