# Supplementary material for: DNA Shuffling of aprE Genes to Increase Fibrinolytic Activity and Thermostability
Source: J Microbiol Biotechnol. 2022 Apr 25;32(6):800–7. doi: 10.4014/jmb.2202.02017 (PMC9628911; doi:10.4014/jmb.2202.02017)
Supplement: Supplementary file 1 [file jmb-32-6-800-supple.pdf]

J. Microbiol. Biotechnol.
https://doi.org/10.4014/jmb.2202.02017

Fig. S1. Nucleotide sequence of *aprEFSM4* and alignment with template genes.

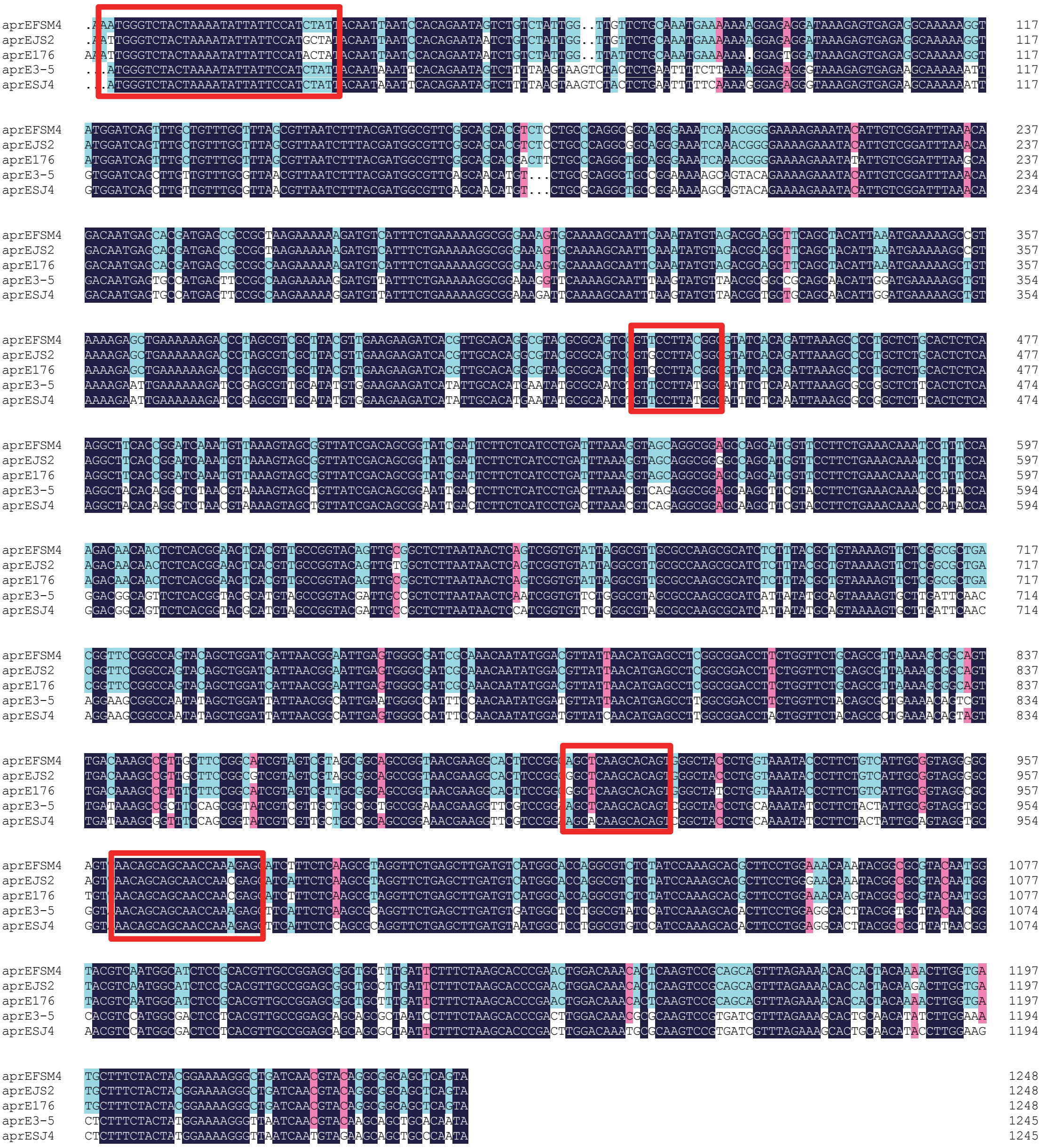

Fig. S2. Amino acid sequence of AprEFM4 and alignment with those of template enzymes.

```
HK176     MRGKKVWISLLFALALIFTMAFGSTTSAQAAGKSNGEKKYIVGFKQTMSTMSAAKKKDVI     60
FSM4      MRGKKVWISLLFALALIFTMAFGSTSPAQAAGKSNGEKKYIVGFKQTMSTMSAAKKKDVI     60
JS2       MRGKKVWISLLFALALIFTMAFGSTSPAQAAGKSNGEKKYIVGFKQTMSTMSAAKKKDVI     60
SJ4       MRSKKLWISLLFALTLIFTMAFSN-MSAQAAGKSSTEKKYIVGFKQTMSAMSSAKKKDVI     59
CH3-5     MRSKKLWISLLFALTLIFTMAFSN-MSAQAAGKSSTEKKYIVGFKQTMSAMSSAKKKDVI     59
          ** ** ******** *******     *******  ************* ** *******

HK176     SEKGGKVQKQFKYVDAASATLNEKAVKELKKDPSVAYVEEDHVAQAYAQSVPYGVSQIKA     120
FSM4      SEKGGKVQKQFKYVDAASATLNEKAVKELKKDPSVAYVEEDHVAQAYAQSVPYGVSQIKA     120
JS2       SEKGGKVQKQFKYVDAASATLNEKAVKELKKDPSVAYVEEDHVAQAYAQSVPYGVSQIKA     120
SJ4       SEKGGKIQKQFKYVNAAAATLDEKAVKELKKDPSVAYVEEDHIAHEYAQSVPYGISQIKA     119
CH3-5     SEKGGKVQKQFKYVNAAAATLDEKAVKELKKDPSVAYVEEDHIAHEYAQSVPYGISQIKA     119
          ****** ******* ** *** ******************** *  ******** *****

HK176     PALHSQGFTGSNVKVAVIDSGIDSSHPDLKVAGGASMVPSETNPFQDNNSHGTHVAGTVA     180
FSM4      PALHSQGFTGSNVKVAVIDSGIDSSHPDLKVAGGASMVPSETNPFQDNNSHGTHVAGTVA     180
JS2       PALHSQGFTGSNVKVAVIDSGIDSSHPDLKVAGGASMVPSETNPFQDNNSHGTHVAGTVV     180
SJ4       PALHSQGYTGSNVKVAVIDSGIDSSHPDLNVRGGASFVPSETNPYQDGSSHGTHVAGTIA     179
CH3-5     PALHSQGYTGSNVKVAVIDSGIDSSHPDLNVRGGASFVPSETNPYQDGSSHGTHVAGTIA     179
          ******* ********************* * **** ******* **  *********

HK176     ALNNSVGVLGVAPSASLYAVKVLGADGSGQYSWIINGIEWAIANNMDVINMSLGGPSGSA     240
FSM4      ALNNSVGVLGVAPSASLYAVKVLGADGSGQYSWIINGIEWAIANNMDVINMSLGGPSGSA     240
JS2       ALNNSVGVLGVAPSASLYAVKVLGADGSGQYSWIINGIEWAIANNMDVINMSLGGPSGSA     240
SJ4       ALNNSIGVLGVAPSASLYAVKVLDSTGSGQYSWIINGIEWAISNNMDVINMSLGGPTGST     239
CH3-5     ALNNSIGVLGVAPSASLYAVKVLDSTGSGQYSWIINGIEWAISNNMDVINMSLGGPSGST     239
          ***** *****************   **************** ************* **

HK176     ALKAAVDKAVSSGIVVVAAAGNEGTSGGSSTVGYPGKYPSVIAVGAVNSSNQRASFSSVG     300
FSM4      ALKAAVDKAVASGIVVVAAAGNEGTSGSSSTVGYPGKYPSVIAVGAVNSSNQRASFSSVG     300
JS2       ALKAAVDKAVASGVVVVAAAGNEGTSGGSSTVGYPGKYPSVIAVGAVNSSNQRASFSSVG     300
SJ4       ALKTVVDKAVSSGIVVAAAAGNEGSSGSTSTVGYPAKYPSTIAVGAVNSSNQRASFSSAG     299
CH3-5     ALKTVVDKAASSGIVVAAAAGNEGSSGSSSTVGYPAKYPSTIAVGAVNSSNQRASFSSAG     299
          ***  ****  ** ** ******* **  ****** **** ***************** *

HK176     SELDVMAPGVSIQSTLPGNKYGAYNGTSMASPHVAGAAALILSKHPNWTNTQVRSSLENT     360
FSM4      SELDVMAPGVSIQSTLPGNKYGAYNGTSMASPHVAGAAALILSKHPNWTNTQVRSSLENT     360
JS2       SELDVMAPGVSIQSTLPGNKYGAYNGTSMASPHVAGAAALILSKHPNWTNTQVRSSLENT     360
SJ4       SELDVMAPGVSIQSTLPGGTYGAYNGTSMATPHVAGAAALILSKHPTWTNAQVRDRLEST     359
CH3-5     SELDVMAPGVSIQSTLPGGTYGAYNGTSMATPHVAGAAALILSKHPTWTNAQVRDRLEST     359
          ******************  ********** *************** *** ***  ** *

HK176     TTKLGDAFYYGKGLINVQAAAQ     382
FSM4      TTKLGDAFYYGKGLINVQAAAQ     382
JS2       TTRLGDAFYYGKGLINVQAAAQ     382
SJ4       ATYLGSSFYYGKGLINVEAAAQ     381
CH3-5     ATYLGNSFYYGKGLINVQAAAQ     381
           * **  ********** ****
```
